# Evaluation of gut microbiota of iranian patients with celiac disease, non-celiac wheat sensitivity, and irritable bowel syndrome: are there any similarities?

**DOI:** 10.1186/s12876-023-02649-y

**Published:** 2023-01-16

**Authors:** Kaveh Naseri, Hossein Dabiri, Meysam Olfatifar, Mohammad Amin Shahrbaf, Abbas Yadegar, Mona Soheilian-Khorzoghi, Amir Sadeghi, Saeede Saadati, Mohammad Rostami-Nejad, Anil K. Verma, Mohammad Reza Zali

**Affiliations:** 1 School of Health and Biomedical Sciences, RMIT University, Melbourne, VIC Australia; 2 Department of Microbiology, School of Medicine, Shahid Beheshti University of Medical Sciences, Tehran, Iran; 3 Gastroenterology and Hepatology Diseases Research Center, Qom University of Medical Sciences, Qom, Iran; 4 Celiac Disease Department, Gastroenterology and Liver Diseases Research Center, Research Institute for Gastroenterology and Liver Diseases, Shahid Beheshti University of Medical Sciences, Tehran, Iran; 5 Foodborne and Waterborne Diseases Research Center, Research Institute for Gastroenterology and Liver Diseases, Shahid Beheshti University of Medical Sciences, Tehran, Iran; 6 Celiac Disease Research Laboratory, Department of Pediatrics, Università Politecnica Delle Marche, 60123 Ancona, Italy

**Keywords:** Celiac disease, Irritable bowel syndrome, Non-celiac wheat sensitivity, Gut microbiota, Dysbiosis

## Abstract

**Background and aims:**

Individuals with celiac disease (CD), non-celiac wheat sensitivity (NCWS), and irritable bowel syndrome (IBS), show overlapping clinical symptoms and experience gut dysbiosis. A limited number of studies so far compared the gut microbiota among these intestinal conditions. This study aimed to investigate the similarities in the gut microbiota among patients with CD, NCWS, and IBS in comparison to healthy controls (HC).

**Materials and methods:**

In this prospective study, in total 72 adult subjects, including CD (n = 15), NCWS (n = 12), IBS (n = 30), and HC (n = 15) were recruited. Fecal samples were collected from each individual. A quantitative real-time PCR (qPCR) test using 16S ribosomal RNA was conducted on stool samples to assess the relative abundance of *Firmicutes*, *Bacteroidetes*, *Bifidobacterium* spp., and *Lactobacillus* spp.

**Results:**

In all groups*, Firmicutes* and *Lactobacillus *spp. had the highest and lowest relative abundance respectively. The phylum *Firmicutes* had a higher relative abundance in CD patients than other groups. On the other hand, the phylum *Bacteroidetes* had the highest relative abundance among healthy subjects but the lowest in patients with NCWS*.* The relative abundance of *Bifidobacterium *spp. was lower in subjects with CD (*P* = 0.035) and IBS (*P* = 0.001) compared to the HCs. Also, the alteration of *Firmicutes* to *Bacteroidetes* ratio (F/B ratio) was statistically significant in NCWS and CD patients compared to the HCs (*P* = 0.05).

**Conclusion:**

The principal coordinate analysis (PCoA), as a powerful multivariate analysis, suggested that the investigated gut microbial profile of patients with IBS and NCWS share more similarities to the HCs. In contrast, patients with CD had the most dissimilarity compared to the other groups in the context of the studied gut microbiota.

**Supplementary Information:**

The online version contains supplementary material available at 10.1186/s12876-023-02649-y.

## Introduction

The human gastrointestinal (GI) tract harbors an incredibly complex and abundant ensemble of microbes referred to as gut microbiota [1]. Gut microbiota plays a pivotal role in human health and diseases [2–4] and its composition depends on various factors, including age [5], diet [6], geography [7], malnourishment [8], race, ethnicity [9], and socioeconomic status [10]. Balance in the gut microbiota composition and the presence or absence of critical species capable of causing specific responses contribute to ensuring homeostasis in the intestinal mucosa and other organs [11–14]. An imbalanced or disturbed composition and quantity of the gut microbiota, known as dysbiosis [15], can affect the bacterial function and is associated with a variety of GI disorders [16–20]. Celiac disease (CD), non-celiac wheat sensitivity (NCWS), and irritable bowel syndrome (IBS), have intestinal dysbiosis as a causative factor in the initiation of their symptoms [21–24]. CD is a chronic small intestinal inflammation, triggered by the consumption of gluten, resulting in villous atrophy in genetically susceptible individuals [25]. IBS is a functional gastrointestinal disorder that afflicts nearly 15% of the population worldwide, characterized by recurrent abdominal pain or discomfort, and changes in bowel habits, in the absence of any other disease to cause these symptoms [26, 27]. NCWS is still an unclear diagnosis, characterized by a combination of CD-like or IBS-like symptoms (e.g., diarrhea, abdominal pain, bloating), behavior disturbances, and systemic manifestations, related to the ingestion of gluten in subjects who are not affected by either CD or wheat allergy [28, 29]. Therefore, since these three disorders are related to dysbiosis in gut microbiota and share similarities in their symptoms, these data form a hypothesis regarding the possible similarities in the alterations of the gut microbiota in subjects with the aforementioned disorders. Although the findings are inconsistent, previous studies mainly reported decreased levels of fecal *Lactobacilli* and *Bifidobacteria*, and increased ratios of *Firmicutes* to *Bacteroidetes* in patients with IBS when compared to healthy individuals [21, 30–32]. According to most studies conducted on the gut microbiota of CD patients, *Bifidobacteria* and *Lactobacilli* levels are decreased in comparison to healthy controls [22, 33, 34]. Due to NCWS being a relatively new diagnosis, few studies have examined gut microbiota in this group.

To the best of our knowledge, no previous studies have investigated the possible similarities in the gut microbiota profile of patients with CD, NCWS, and IBS compared to healthy control. Hence, we designed this monocentric prospective observational study to compare the relative abundance of *Firmicutes* and *Bacteroidetes*, as the two most dominant phyla [35–38], and *Bifidobacterium* and *Lactobacillus,* as two highly controversial genera of fecal microbial communities, among Iranian subjects with CD, NCWS, and IBS compared to HCs.

## Materials and methods

### Study population

From March 2020 to November 2020, consecutive newly diagnosed CD, NCWS, and IBS patients were recruited from an outpatient gastroenterology clinic in Taleghani Hospital, Tehran, Iran. Convenience sampling was used for participants’ selection. Subjects who had recently been diagnosed with CD, NCWS, and IBS, and were not on therapeutic diets such as gluten-free or low-FODMAP diets or taking supplements such as probiotics, prebiotics, or synbiotics were considered as patients groups. CD diagnosis was established according to the "4 out of 5" rule and four of the following criteria were considered sufficient for disease diagnosis: typical CD related symptoms, positivity of CD-specific antibodies, HLA-DQ2 or 8 genotypes, intestinal damages at duodenal biopsy and clinical response to GFD [39]**.** Twelve patients with NCWS that fulfilled the Salerno consensus criteria [40] were included. All NCWS subjects demonstrated negative serology results for tissue-transglutaminase IgA antibodies, and the duodenal biopsy results were normal [41].

IBS diagnosis was based on fulfilling the ROME-IV criteria [27], including recurrent abdominal pain at least one day per week over the previous 3 months, along with two or more of the following criteria: (a) changes in defecation, (b) changes in frequency, and (c) changes in the form of stool, with no medication to alleviate symptoms in the last 3 months. Anti-Tissue Trans-glutaminase (Anti-tTG) and/or endomysial antibodies (EMA), histological findings compatible with atrophy (according to the Marsh classification), and wheat-specific Immunoglobulin E (IgE) levels were negative in all thirty patients with IBS. Apart from these, fifteen healthy volunteers, with no history of digestive pathologies lacking CD-specific antibodies, were enrolled in the healthy control (HC) group. These HCs had normal bowel movements without abdominal symptoms, coronary artery disease, inflammatory conditions, IBS, NCWS, and diabetes mellitus.

Pregnant and lactating women, individuals with any systemic inflammatory diseases like autoimmune conditions, gastrointestinal diseases (i.e. inflammatory bowel disease (IBD)) or any other acute or chronic diseases, gastrointestinal surgery, cancer, and those who were not willing to participate in the study were excluded from all study groups. Non-steroidal anti-inflammatory drugs (NSAIDs) usage, excessive alcohol consumption, systemic use of immunosuppressive agents, poorly controlled psychiatric diseases and the history of broad-spectrum antibiotics and probiotics consumption were also considered as exclusion criteria. Participants were also asked not to take any antibiotics, eat spicy food, and smoke four weeks prior to sample collection.

### Fecal samples collection and homogenization

Fresh early-morning fecal samples, representative of whole gut microbiome, were collected from each participant in sterile fecal specimen containers at the study's baseline. A water ban was also required after midnight and before collecting the samples in the morning. Stool specimens were collected and handled by experienced clinicians and trained technicians. Homogenization of the stool samples was conducted through agitation by using a vortex. Afterward, stool samples were divided into three aliquots within 3 h of defecation. Using screw-capped cryovial containers, the aliquots were immediately frozen and stored at − 80 °C until used for DNA extraction [42].

### DNA extraction from fecal samples

QIAamp® DNA Stool Mini Kit (Qiagen Retsch GmbH, Hannover, Germany) was used for DNA extraction [43]. DNA concentration was quantified by NanoDrop ND-2000 Spectrophotometer (NanoDrop products, Wilmington, DE, USA). In addition, Nanodrop (DeNovix Inc., USA) was used for assessing the concentration and purity of the extracted DNA. Extracted DNA samples were stored at − 20 °C until further analysis.

### Microbiota analysis by quantitative real-time PCR (qPCR)

We performed qPCR assay to evaluate the relative abundance of two bacterial phyla, including *Firmicutes* and *Bacteroidetes*, and two genera, including *Bifidobacterium* spp. and *Lactobacillus* spp. The qPCR was conducted by SYBR Green chemistry using universal and group-specific primers based on the bacterial 16S rRNA sequences presented in Additional file 1: Table S1. All PCRs were performed in a volume of 25 μL, comprising 12.5 μL of SYBR green PCR master mix (Ampliqon, Odense, Denmark), 1 μL of 10 pmol of forward, and reverse primers, and 100 ng of the DNA template.

Rotor-Gene® Q (Qiagen, Germany) real-time PCR system was used for the PCR amplification. The amplification reaction parameters were assumed as 95 °C for 10 min and 40 cycles at 95 °C for 20 and 30 s for each primer (Additional file 1: Table S1) and 72 °C for the 20 s. Melting curve analysis was conducted to assess the amplification accuracy by increasing temperature from 60 to 95 °C (0.5 °C increase in every 5 s). The relative abundance of studied taxa was evaluated based on the ratio of the 16S rRNA copy number of the specific bacteria to the total 16S rRNA copy number of all bacteria using the previously described method [32]. Accordingly, the average Ct value for primers was reported as the percentage values using the following formula:

$$X = \frac{{\left( {{\text{Eff}}.{\text{ Univ}}} \right)^{{\text{Ct univ}}} }}{{\left( {{\text{Eff}}.{\text{ Spec}}} \right)^{{\text{Ct spec}}} }} \times {1}00$$

The percentage of 16S taxon-specific copy numbers was indicated by “X”. Furthermore, “Eff. Univ” and “Eff. Spec” represents the efficiency of the universal primers (2 = 100% and 1 = 0%) and the efficiency of the taxon-specific primers respectively. The threshold cycles registered by the thermocycler were indicated by “Ct univ” and “Ct spec”.

### Statistical analysis

Analysis of collected data was performed using Statistical Package for the Social Sciences (SPSS) version 25.0, SPSS Inc., Chicago, IL, USA. Figures were drawn using GRAPHPAD Prism 8.4.0 (GraphPad Software, Inc, San Diego, CA). Quantitative variables were reported as mean ± standard deviation (SD) and qualitative variables were reported as numerical (%) data. ANOVA test was used for the assessment of the relative abundance differences between the two phyla. In addition, we used R software and Principal Coordinate Analysis (PCoA) method to assess dissimilarities in this study. The PCoA was calculated based on the Bray Curtis dissimilarity method [44].

## Results

### Demographics

Seventy-two samples from adult participants were enrolled in this study. Due to age-related changes in the gut microbiota, the study groups were adjusted according to their age so as not to have significant differences between them (*P* = 0.76). Thirty-three patients were male (45.8%), and the mean age of the patients was 35.5 ± 6.4. Fifteen patients (20.8%) were in the HC group, 30 (41.7%) in the IBS group, 12 (16.6%) in the NCWS group, and 15 (20.8%) in the CD group. The baseline characteristics of the patients are presented in Table 1.

**Table 1:** Baseline characteristics of study participants at enrollment

Variables	HC (n = 15)	IBS (n = 30)	NCWS (n = 12)	CD (n = 15)	Total (n = 72)	*P*-value*
Age (years)	32.8 ± 12.2	37.8 ± 10.7	31.8 ± 6.4	40.1 ± 8.2	35.5 ± 6.4	0.76
Males (n%)	7 (46.7%)	15 (50%)	5 (41.7%)	6 (50%)	33 (45.8%)	0.83
Females (n%)	8 (53.3%)	15 (50%)	7 (58.3%)	6 (50%)	39 (54.2%)	0.45
Smoking (n%)	4 (26.6%)	9 (30%)	4 (33.3%)	2 (13.3%)	19 (26.4%)	0.65

HC, healthy control; IBS, irritable bowel syndrome; NCWS, non-celiac wheat sensitivity; CD, celiac disease

*P-values obtained by Kruskal–Wallis test

### Microbiota relative abundance analysis

**Fig. 1:**
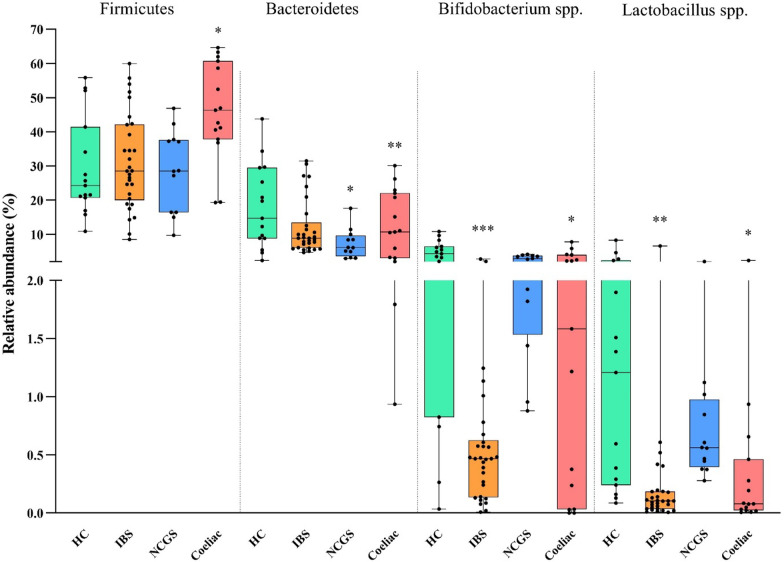
Box plot for the distribution of the selected bacterial taxa by the median abundance that constitutes the fecal microbiota in each group of the study population. Differences in each group of the patients were compared to the healthy control (HC) and were considered to be statistically significant when *P < 0.05, **P < 0.01, and ***P < 0.001

Significant changes in the gut microbiota composition across various groups have been observed. The relative abundance analysis indicated that *Firmicutes* was the most abundant bacterial group and the predominant phylum in all the studied groups (HC: 29.5 ± 13.9%, IBS: 31.2 ± 13.6%, NCWS: 28.6 ± 11.4%, and CD: 46.2 ± 14.0%). At the same time, *Bifidobacterium *spp. was the dominant genus among the studied participants, with the highest relative abundance in patients with HCs (4.4 ± 3.3%). According to our findings, patients with CD had a higher relative abundance of the phylum *Firmicutes* than the other groups, including the HC group, for which this difference was statistically significant (*p* = 0.002). Whereas the phylum *Bacteroidetes* was significantly lower in patients with IBS (*P* = 0.049) and NCWS (*P* = 0.006). This phylum had the lowest relative abundance in the NCWS group (7.3 ± 4.0%). In addition, the relative abundance of *Bifidobacterium *spp. was statistically lower in subjects with CD (*P* = 0.022) and IBS (*P* = 0.001); with the lowest percentage in the IBS group (0.5 ± 0.5). Moreover, *Lactobacillus* spp. was significantly lower in subjects with CD (*P* = 0.022) and IBS (*P* = 0.007) compared to the HCs. The relative abundance of this genus was also lower in subjects with NCWS, though not statistically significant (*P* = 0.12). The results for the relative abundance are presented in Table 2 and Fig. 1. As shown in Table 2 the results obtained from the Kruskal–Wallis test also revealed significant inter-groups differences for all the studied bacteria (p˂0.05).

**Table 2:** The mean of the relative abundance for taxonomical groups in each group of the study participants

Taxonomical Group	HC (n = 15)	IBS (n = 30)	NCWS (n = 12)	CD (n = 15)	*P*-value^*^
*Firmicutes*	29.5 ± 13.9^¥^	31.2 ± 13.6	28.6 ± 11.4	46.2 ± 14.0	0.0022
*Bacteroidetes*	18.0 ± 11.9	12.0 ± 7.9	7.3 ± 4.0	12.4 ± 9.5	0.028
*Bifidobacterium *spp.	4.4 ± 3.3	0.5 ± 0.5	2.6 ± 1.1	2.1 ± 2.3	0.001
*Lactobacillus *spp.	1.7 ± 2.1	0.3 ± 1.1	0.7 ± 0.4	0.3 ± 0.6	0.009

HC, healthy control; IBS, irritable bowel syndrome; NCWS, non-celiac wheat sensitivity; CD, celiac disease

*P-values represent the intergroup differences using the Kruskal–Wallis test

^¥^Values are presented as mean ± SD

### Firmicutes to Bacteroidetes ratio

**Fig. 2:**
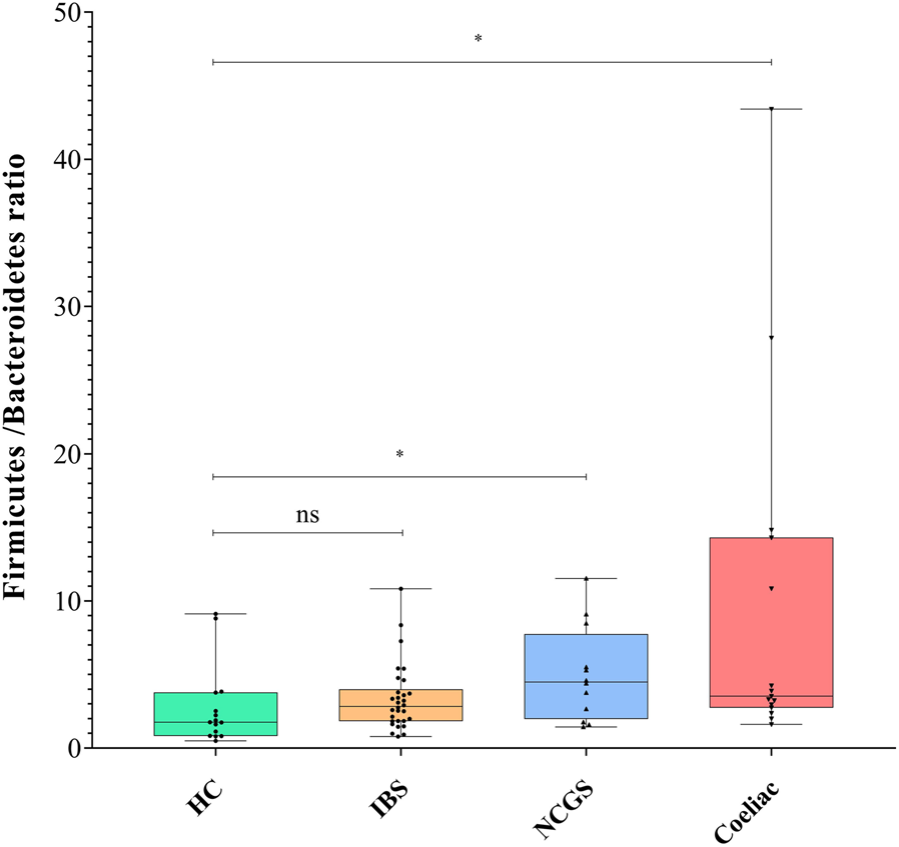
Box plots showing the Firmicutes to Bacteroidetes (F/B) I each group of participants. This ratio was significantly (*P = 0.05) increased in the NCWS and CD patients but non-significant in the IBS patients compared with the healthy controls (HC)

The ratio of *Firmicutes* to *Bacteroidetes* (F/B ratio) was significantly higher in patients with NCWS and CD than the HC individuals (*P* = 0.05). However, F/B ratio was not statistically different between subjects with IBS and the HCs. The results of the F/B ratio analysis are illustrated in Fig. 2.

### Dissimilarity and principal coordinate analysis (PCoA)

**Fig. 3:**
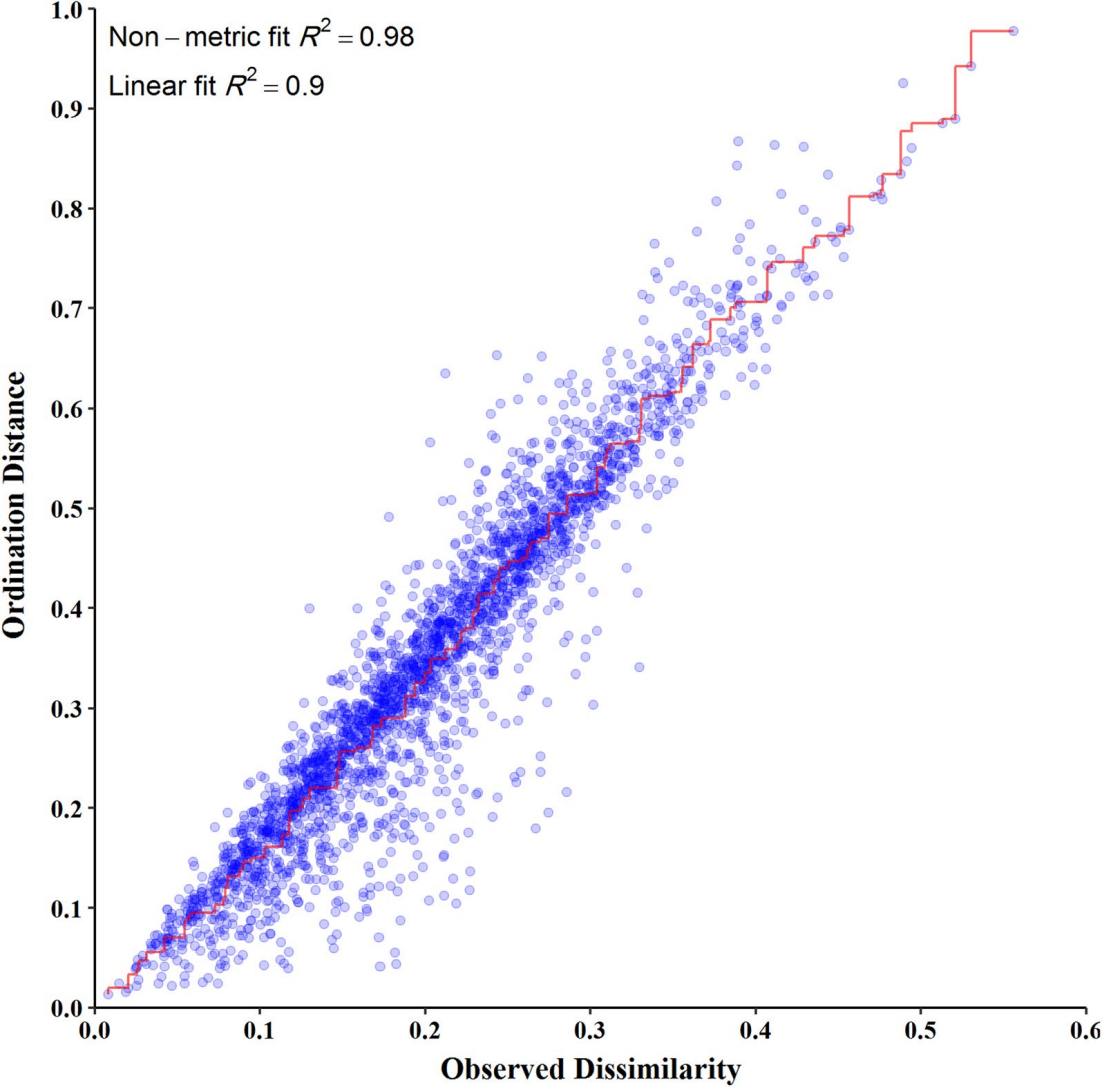
Shepherd plot showing the correlation between the distance from the dissimilarity matrix and the coordination distance for NMDS analysis

**Fig. 4:**
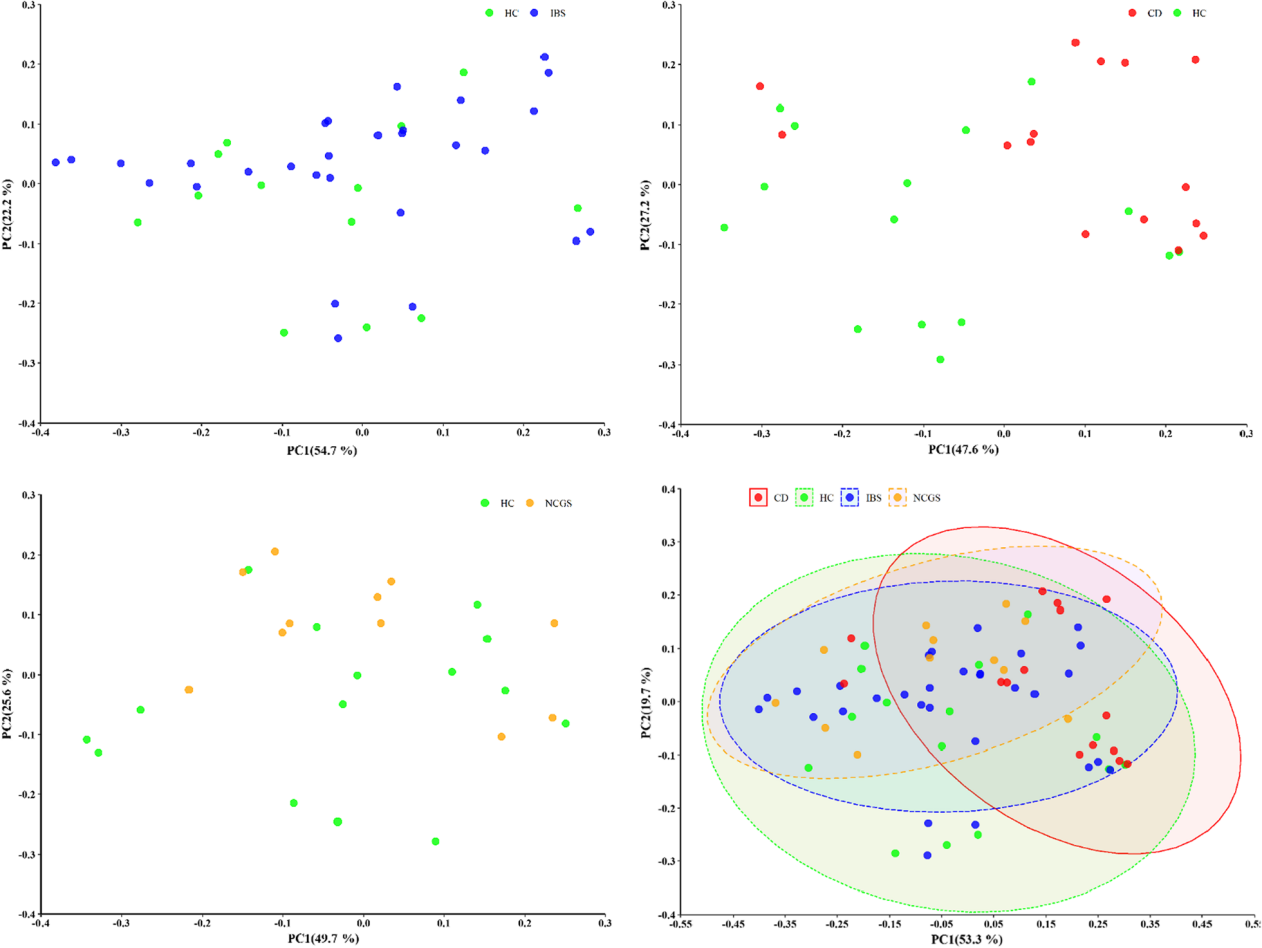
Bray–Curtis dissimilarity metric plotted in PCoA space comparing the microbial communities from different patient groups (CD, NCWS, IBS, and HC). Each circle representing a participant colored according to the studied group

We measured the extent of fit of the ordination by plotting the observed dissimilarity (as calculated by the dissimilarity matrix) to the ordination distance using a shepherd plot, which yielded an R^2^ = 0.996, indicating a good fit between the ordination distance and the observed dissimilarity, as calculated by Bray–Curtis index (Fig. 3). The dissimilarity between the microbiome of different groups is shown in Fig. 4. The PCoA suggests that IBS and NCWS patients share more gut microbiota similarities with HCs. In contrast, CD patients had the highest level of dissimilarity compared to the other groups.

## Discussion

The current study examined fecal samples from adult participants with three GI disorders, including CD, NCWS, and IBS. Comparing gut dysbiosis to healthy controls, the microbiota analysis interestingly showed a significant difference in the relative abundance of *Firmicutes, Bacteroidetes, Bifidobacterium* spp., and *Lactobacillus *spp. in CD patients. In addition, the analysis of the relative abundance of *Bifidobacterium *spp. and *Lactobacillus *spp. in IBS patients and *Bacteroidetes* in NCWS revealed a statistically significant decrease compared to the HC group. Furthermore, *Firmicutes* to *Bacteroidetes* ratio (F/B ratio) assessment, as a valuable index for detecting the alterations in gut microbiota, was another aim of the current study. Changes in the F/B ratio could be particularly important. *Firmicutes* and *Bacteroidetes* are two predominant phyla accounting for up to 90% of the total gut microbiota composition [45]. The F/B ratio has been suggested as an important index of gut microbiota health. [46]. This ratio is associated with different pathological states [47]. For instance, the association of a high F/B ratio with several conditions including GI disorders has been observed repeatedly [48–50]. Particularly, it is associated with the production of short-chain fatty acids such as butyrate and propionate [51]. Short-chain fatty acids generated by microbiota can have a significant influence on human health. The anti-inflammatory molecule butyrate, in particular, acts both on enterocytes and circulating immune cells, regulating gut barrier integrity. Additionally, propionate production plays a crucial role in human health since it promotes satiety and prevents hepatic lipogenesis, which in turn lowers cholesterol production [52, 53]. Moreover, the increased F/B ratio is associated with an increased energy harvest from colonic fermentation [54]. According to our analysis, the F/B ratio was significantly higher in the subjects with CD and NCWS than in the HCs. In contrast, it was not statistically significant in subjects with IBS, suggesting a higher level of alteration in the gut microbiota of individuals with CD and NCWS than in the IBS compared to the HCs. Recent studies suggested that the alteration of gut microbiota composition is associated with CD pathogenesis [55–57]. In the study of Golfetto et al., the concentration of *Bifidobacterium *spp. in CD patients was significantly lower compared to the HCs [58]. Another study conducted by Bodkhe et al., reported that *Firmicutes* and *Bacteroidetes* were the major phyla in the duodenal microbiota of subjects with CD [59]. Several other studies have demonstrated that *Bifidobacterium *spp. and *Lactobacillus *spp. protect the intestinal epithelial cells from gliadin damage [60–62]. Accordingly, it has been suggested that the fecal transplant which can cause an increment in *Bifidobacterium *spp. could reverse the inflammatory pathway in CD patients [63]. Among all the groups we studied, *Firmicutes* predominated the gut microbiota. In addition, *Bacteroidetes*, *Bifidobacterium *spp.*,* and *Lactobacillus *spp. had significantly lower abundance in subjects with CD compared to the HCs. In terms of the alteration and relative abundance of the studied bacterial groups, the current study's results were largely consistent with the previous reports.

Gut microbiota dysbiosis in individuals with IBS has been reported in several studies [64–66]. In fact, gastrointestinal dysbiosis in these patients is associated with intestinal hypersensitivity, mucosal immune activation, and chronic inflammation, which are the three important pathophysiological factors in this disease [67, 68]. A number of studies have reported lower amounts of *Bacteroidetes* and higher amounts of *Firmicutes* in subjects with IBS compared to HCs [32, 69, 70]. In the current study, both of these phyla had lower relative abundances than those of HCs, although their differences were not statistically significant. Furthermore, it has been suggested that IBS is associated with the lower relative abundance of *Bifidobacterium* spp. and *Lactobacillus* spp. [71, 72] which is in accordance with the current study. However, it is noteworthy that Maccaferri et al. observed an increase in the relative abundance of *Bifidobacterium* spp. and *Lactobacillus* among subjects with IBS [73]. It seems that further evidence is needed to confirm these results. As for NCWS, dysbiosis in these individuals is one of the important issues which can cause constipation, diarrhea, chronic inflammation, intestinal hypersensitivity, and immune dysfunction [74]. Garcia-Mazcorro et al. reported a high relative abundance of *Firmicutes* and a low relative abundance of *Bacteroidetes* in the fecal microbiota of the individuals with NCWS [75]. According to the current study, the Phylum Bacteroidetes was significantly lower in NCWS patients compared to HCs, in agreement with the previous study.

Analysis of the dissimilarity and PCoA in this study suggests that individuals with CD experience a higher level of dysbiosis compared to the other subjects with microbiota-related GI disorders. In fact, fewer similarities were observed in the studied bacterial profile of subjects with IBS and those with NCWS. Overall, it may explain why this disorder exhibits more severe symptoms when compared to the other GI disorders, suggesting that the recovery of gut microbiota should be emphasized more in the treatment of this disease. According to these analyses, the composition of the gut microbiota in the subjects with IBS and NCWS is more similar to that of the HCs’, which may suggest a more favorable outcome for IBS and NCWS than for CD.

The present study had some limitations. First, the sample size is not large enough to extrapolate the results. Actually, the present study has monocentric nature that was conducted in a limited population with specific features. Even if this matter has been addressed with bigger sample sizes, the results cannot be generalized from one population to others. Second, based on the meta-genomic data, the human gut microbiome may contain more than 1000 bacterial species. Although the studied bacterial phyla and genera are the most dominant and critical taxonomical groups, there are other groups that should be taken into consideration. Third, alimentary habits of the included subjects, which can consistently modify gut microbiota, were not assessed in the current study. Considering the fact that, eating habits such as using fiber sub-types, food additives, ultra-processed foods and etc. can affect the gut bacteria composition, performing further similar microbiota studies evaluating patients’ dietary pattern is highly recommended. Moreover, the lack of a follow-up of patients and comparison of results before and after receiving treatment is another important limitation.

To our knowledge, no previous publication has compared the gut microbiota profile of subjects with CD, NCWS, and IBS. In fact, the potential overlap between NCWS and IBS diagnosis and the unavailability of gluten challenge tests in many medical centers make it difficult to explore the gut microbiota among these groups. Thus, this study represents promising findings for future research. Additionally, investigating all components of the gut microbiota including bacteria, viruses, fungi, and archaea in order to identify microbial patterns, conducting multi-centric studies, and examining the fecal microbiome and mucosal microbiome simultaneously to have a better perspective on the differences between the mucosal microbiome and fecal microbiome would have been of great importance.

## Conclusion

Results of our study indicate that the human intestinal microbiota composition differs across the studied groups with different microbiota-related GI disorders. Specifically, patients with CD had the highest level of dissimilarity compared to the other studied groups with GI disorders and HCs. In contrast, those with IBS had the lowest level of dissimilarity with HCs. This study found some microbial changes that were inconsistent with the previous results, possibly due to genetics, geographical pattern, ethnicity, or diet.

## Supplementary Information

**Additional file 1: Supplementary Table 1.** The taxon-specific primers used in this study.

## Data Availability

The datasets used and/or analysed during the current study available from the corresponding author on reasonable request.
