# Supplementary material for: Evaluation of gut microbiota of iranian patients with celiac disease, non-celiac wheat sensitivity, and irritable bowel syndrome: are there any similarities?
Source: BMC Gastroenterol. 2023 Jan 16;23:15. doi: 10.1186/s12876-023-02649-y (PMC9841652; doi:10.1186/s12876-023-02649-y)
Supplement: Supplementary file 1 — Additional file 1: Supplementary Table 1. The taxon-specific primers used in this study. [file 12876_2023_2649_MOESM1_ESM.doc]

**Supplementary Table 1.** The taxon-specific primers used in this study.

| **Target taxon** | **Primer name** | **Primer sequence (5ꞌ-3ꞌ)** | **Amplicon length (bp)** | **Reference** |
| --- | --- | --- | --- | --- |
| Eubacteria | UniF340  UniR514 | ACTCCTACGGGAGGCAGCAGT  ATTACCGCGGCTGCTGGC | ~ 200 bp | [1] |
| *Lactobacillus spp.* | Lacto-F  Lacto-R | TGGATGCCTTGGCACTAG  AAATCTCCGGATCAAAGCTTAC | ~ 89 bp | [2] |
| *Bifidobacterium spp.* | Bifid-F  Bifid-R | GGGATGCTGGTGTGGAAGAG  TGCTCGCGTCCACTATCCAG | ~ 200 bp | [2] |
| *Bacteroidetes* | Bac960-F  Bac1100-R | GTTTAATTCGATGATACGCG  TTAAGCCGACACCTCACG | ~ 137 bp | [3] |
| *Firmicutes* | Firm934-F  Firm1060-R | GGAG**Y**ATGTGGTTTAATTCGAAGCA  AGCTGACGACAACCATGCAC | ~ 129 bp | [3] |

The nucleotides in bold type represent: Y, C or T; K, G or T; M, A or C; R, A or G; W, A or T.

**References:**

1. Moraes JG, Motta ME, Beltrão MF, Salviano TL, Silva GA. Fecal microbiota and diet of children with chronic constipation. International journal of pediatrics. 2016 Jun 23;2016.

2. Wang IK, Lai HC, Yu CJ, Liang CC, Chang CT, Kuo HL, Yang YF, Lin CC, Lin HH, Liu YL, Chang YC. Real-time PCR analysis of the intestinal microbiotas in peritoneal dialysis patients. Applied and environmental microbiology. 2012 Feb 15;78(4):1107-12.

3. Matsuki T, Watanabe K, Fujimoto J, Takada T, Tanaka R. Use of 16S rRNA gene-targeted group-specific primers for real-time PCR analysis of predominant bacteria in human feces. Applied and environmental microbiology. 2004 Dec 1;70(12):7220-8.
